# Murine Beta-Amyloid (1–42) Oligomers Disrupt Endothelial Barrier Integrity and VEGFR Signaling via Activating Astrocytes to Release Deleterious Soluble Factors

**DOI:** 10.3390/ijms23031878

**Published:** 2022-02-07

**Authors:** Qian Yue, Xinhua Zhou, Zaijun Zhang, Maggie Pui Man Hoi

**Affiliations:** 1 State Key Laboratory of Quality Research in Chinese Medicine, Institute of Chinese Medical Sciences, University of Macau, Macao SAR 999078, China; yb97528@um.edu.mo (Q.Y.); xinhuazhou9901@163.com (X.Z.); 2 Department of Pharmaceutical Sciences, Faculty of Health Sciences, University of Macau, Macao SAR 999078, China; 3 Institute of New Drug Research, Guangdong Province Key Laboratory of Pharmacodynamic Constituents of Traditional Chinese Medicine & New Drug Research, College of Pharmacy, Jinan University, Guangzhou 510632, China; zaijunzhang@163.com

**Keywords:** blood–brain barrier, murine amyloid-beta, endothelial cells, astrocytes, tight junctions, VEGFR2 signaling pathway

## Abstract

Transgenic mouse models of Alzheimer’s disease (AD) overexpress mutations of the human amyloid protein precursor (*APP*) and presenilin-1 (*PSEN1*) genes, which are known causes of amyloid pathology in familial AD. However, animal models for studying AD in the context of aging and age-related co-morbidities, such as blood–brain barrier (BBB) disruptions, are lacking. More recently, aged and progeroid mouse models have been proposed as alternatives to study aging-related AD, but the toxicity of murine amyloid-beta protein (Aβ) is not well defined. In this study, we aimed to study the potential toxicity of murine Aβ on brain endothelial cells and astrocytes, which are important components of the BBB, using mouse brain endothelial cells (bEnd.3) and astrocytes (C8-D1A). Murine-soluble Aβ (1–42) oligomers (sAβO42) (10 µM) induced negligible injuries in an endothelial monolayer but induced significant barrier disruptions in a bEnd.3 and C8-D1A co-culture. Similar results of endothelial perturbation were observed in a bEnd.3 monolayer treated with astrocyte-conditioned medium (ACM) generated by astrocytes exposed to sAβO42 (ACM-sAβO42), while additional exogenous sAβO42 did not cause further damage. Western blot analysis showed that ACM-sAβO42 altered the basal activities of vascular endothelial growth factor receptor 2 (VEGFR2), eNOS, and the signaling of the MEK/ERK and Akt pathways in bEnd.3. Our results showed that murine sAβO42 was moderately toxic to an endothelial and astrocyte co-culture. These damaging effects on the endothelial barrier were induced by deleterious soluble factors released from astrocytes, which disrupted endothelial VEGFR2 signaling and perturbed cell survival and barrier stabilization.

## 1. Introduction

The blood–brain barrier (BBB) consists of a tightly sealed monolayer of brain endothelial cells together with other supporting cells in the neurovascular units, including pericytes, astrocytes, and neurons [1]. Brain endothelial cells are connected by tight junctions (TJ) and adherens junctions (AJ) and the expressions of their junctional proteins (e.g., zonula occludens-1 (ZO-1), claudin-5, occludin, junction adhesion molecules (JAMs), vascular endothelial cadherin (VE-cadherin), platelet endothelial cell adhesion molecule 1 (PECAM-1) and β-catenin) are directly related to the integrity of the BBB [2]. This physiological barrier is a gatekeeper for brain homeostasis, limiting the entry of potentially harmful blood-derived agents, such as plasma-derived components and immune cells, into the central nervous system (CNS), as well as clearing metabolic waste products and potential neurotoxic molecules from parenchyma to the blood. Recently, early dysfunction and/or breakdown of the BBB were associated with Alzheimer’s disease (AD) before the occurrence of neuropathology and cognitive decline [3]. Apart from the pathological hallmarks of amyloid plaques and neurofibrillary tangles, coexisting cerebrovascular lesions and microvascular alterations are often observed in the brains of AD patients [4,5], and accumulating evidence from independent post-mortem human studies have confirmed the breakdown of the BBB in AD patients, showing the presence of endothelial degeneration and loss of TJ (reviewed in [6]). The “two-hit” vascular hypothesis proposes that endothelial dysfunction at the BBB can be the “first hit”, leading to elevated amyloid beta (Aβ) production and accumulation in the CNS (the “second hit”), thus causing neurodegeneration [7]. Accordingly, the alteration in BBB functions likely precedes the progressive and irreversible cognitive decline in AD.

Two main species of Aβ produced are Aβ42 (which mainly contributes to neuronal plaques) and Aβ40 (which contributes to vascular Aβ deposits) [8]. It is reported that soluble oligomeric Aβ42 is more toxic to endothelial cells compared with Aβ40 [9,10,11]. In in vitro BBB models, exogenous human Aβ42 exposure induces characteristic features of BBB dysfunction, such as endothelial cytotoxicity, loss of TJ, and increased permeability in various types of cultured endothelial cells (e.g., human brain microvascular endothelial cells (HBMEC) [12,13,14], human aortic endothelial cells (HAEC) [9], rat brain microvascular endothelial cells (RBMEC) [15,16], bovine pulmonary arterial endothelial cells (BPAEC) [17], reviewed in [11]). More recently, astrocyte-secreted soluble factors have also been shown to play key roles in Aβ-induced BBB damages in a co-culture BBB model using human cell lines [13,14,18]. Under normal conditions, astrocytes exert homeostatic activities that preserve BBB function and integrity by secreting a range of factors to regulate TJ formation and transporters, cover capillaries with astrocytic end-feet, and modulate inflammatory response in response to CNS insults. However, reactive astrocytes can be induced by brain-derived stimuli, resulting in further BBB disruption either by biochemical secretion or structural detachment. In AD, astrogliosis and swollen astrocytic end-feet are often recognized [19]. 

The disruption of BBB function and integrity has also been demonstrated in transgenic mice expressing mutations in human amyloid precursor protein (*APP*) and presenilin-1 (*PSEN1*) genes linked to autosomal dominant AD, manifested as leakage of molecules across the BBB, loss of TJ proteins, and dysregulated expressions of Aβ-transporters (reviewed in [3]). These models strongly suggest an association between Aβ pathology and BBB disruption. However, the overexpression of mutated human genes leading to Aβ overproduction mimics only the rare inherited form of AD (< 1%), while most AD cases are sporadic with no causative single gene defect. Additionally, it was suggested that human Aβ peptides might aggregate differently in the brain of mice [20]. Previous studies reported that high levels of Aβ were detected in physiologically aged mice (15-month-old) in various brain regions [7]. Early symptoms of AD were observed in mice deficient in neprilysin (a major Aβ-degrading enzyme) and ATP binding cassette subfamily C member 1 (ABCC1, a major transporter for Aβ clearance) [20], and the mice developed various aspects of sporadic AD, including astrogliosis and early synaptic degeneration [21]. Given this, it is proposed that aging-dependent AD mouse models should be established to better represent the disease for the study of the aging-dependent pathogenesis of Aβ plaque formation [7,20]. 

Although aging-dependent Aβ pathology has been shown to contribute to BBB dysfunction in mouse models in vivo [20,21], the direct effects of murine Aβ on the BBB cellular components have rarely been investigated. In our present study, we investigated the direct toxicity of murine Aβ on the BBB using murine endothelial cells and astrocytes. We aimed to unravel the contributions of murine Aβ to the dysregulation of BBB cellular interactions. We employed a bEnd.3 (mouse microvascular brain endothelial cells) and C8-D1A (mouse astrocytic cells) co-culture challenged by murine oligomeric Aβ42 (sAβO42) as an in vitro model to mimic the biochemical environment in the brain of aged mice and further investigated the roles of VEGFR2 signaling pathways in the crosstalk between endothelial cells and astrocytes. Our study provided insights for using aged and progeroid mouse models for studying aging-related AD.

## 2. Results

### 2.1. Characterization of Murine Aβ Preparation 

**Figure 1:**
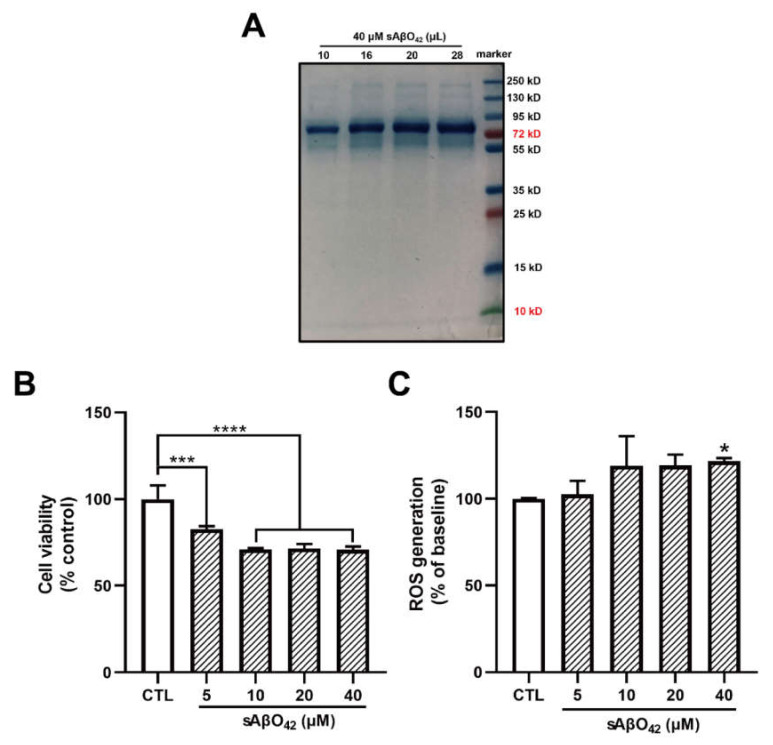
Murine soluble amyloid beta 42 oligomer (sAβO42) exerted negligible and disruptive effects on the barrier function of a bEnd.3 monolayer up to 40 µM. (**A**) Identification for the sizes of the prepared Aβ42 peptides using SDS-PAGE electrophoresis with Coomassie brilliant blue staining. (**B**) The cell viability and (**C**) the relative levels of reactive oxygen species (ROS) in bEnd.3 cells treated with sAβO42 (1–40 µM) for 24 h. Data are shown as means ± SD of at least three independent experiments and were analyzed by one-way ANOVA followed by a post hoc Dunnett’s test. * *p* ≤ 0.1, *** *p* ≤ 0.001, **** *p* ≤ 0.0001 vs. control group.

Accumulating evidence indicates that soluble Aβ oligomers, but not insoluble fibrils or plaques, are linked to the pathogenesis of AD. The majority of soluble Aβ species obtained from the brains of AD patients were oligomers of high molecular weight (HMW) (>70 kDa), while Aβ42 has been shown to induce higher cytotoxicity than Aβ40 [22]. However, transgenic mouse models of AD usually overexpress human *APP* and *PSEN1* mutations that are associated with familial AD but not aging-dependent Aβ pathogenesis. Less is known about mouse Aβ in the mouse brain. Therefore, we investigated the effects of mouse Aβ oligomers on mouse cell lines. We first prepared HMW-soluble Aβ42 oligomers (sAβO42) from synthetic mouse Aβ42, as described [23]. The protein molecular weight of the Aβ preparation was determined using sodium dodecyl sulfate gel electrophoresis (SDS-PAGE) followed by staining with protein-binding dye Coomassie brilliant blue (CCB). Aβ42 was mainly present as HMW oligomers (> 70 kDa) (10–20 mers) in solution (Figure 1A).

### 2.2. Murine sAβO42 Induced Perturbation of Endothelial Junctions and Barrier Integrity in the bEnd.3 Monolayer without Causing Significant Cell Death 

**Figure 2:**
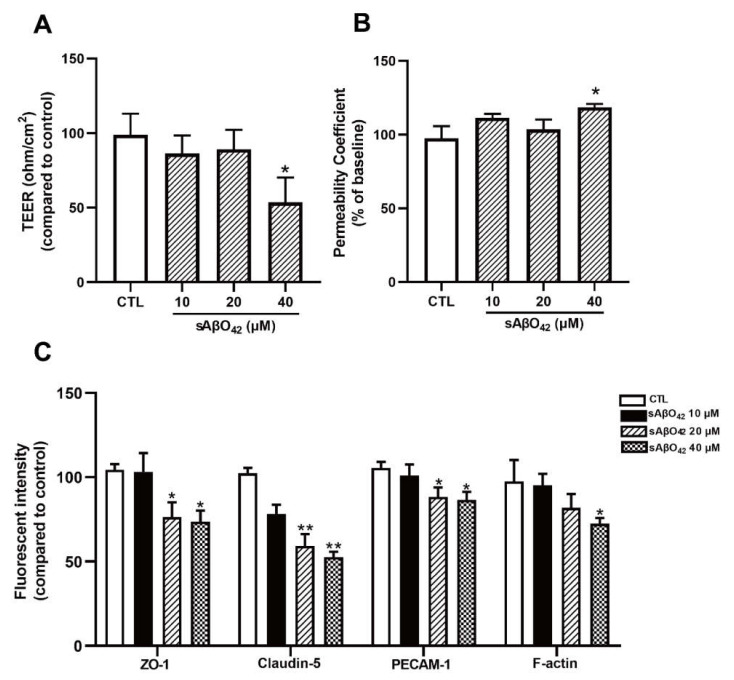
Murine sAβO42 exerted disruptive effects on the barrier function of the bEnd.3 monolayer up to 40µM. (**A**) Transendothelial electrical resistance (TEER) and (**B**) permeability of FITC-dextran across the bEnd.3 monolayer. (**C**) Fluorescent intensity of several junctional proteins (ZO-1, claudin-5, platelet and endothelial cellular adhesion molecule 1 (PECAM-1), and cytoskeleton protein F-actin) in bEnd.3 cells. Data are shown as means ± SD of at least three independent experiments and were analyzed by one-way ANOVA followed by a post hoc Dunnett’s test. * *p* ≤ 0.1, ** *p* ≤ 0.01, vs. control group.

**Figure 3:**
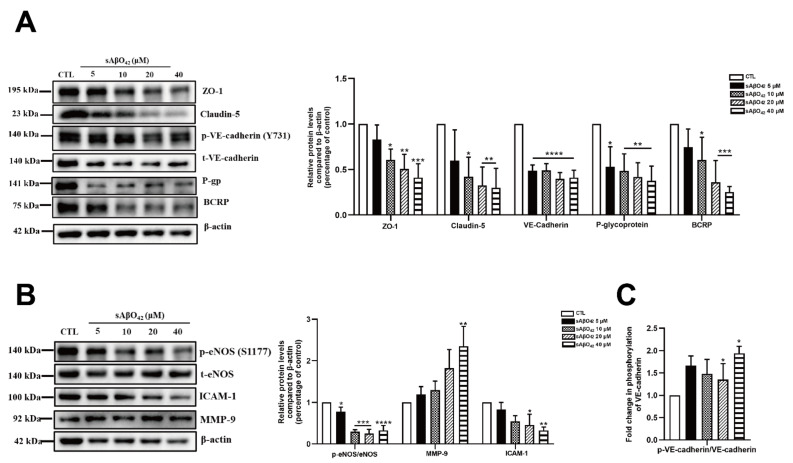
Murine sAβO42 altered the protein levels and activities of constitutively expressed endothelial proteins in the bEnd.3 monolayer. The protein expression levels of junctional proteins (**A**) ZO-1, claudin-5, VE-cadherin, and BBB transporters P-glycoprotein (P-gp) and breast cancer resistance protein (BCRP), as measured by western blot. (**B**) The ratio of phosphorylated endothelial nitric oxide synthase (eNOS)/total eNOS, and the protein expression levels of matrix metallopeptidase 9 (MMP-9) and basal intercellular adhesion molecule 1 (ICAM-1). (**C**) The ratio of phosphorylated endothelial VE-cadherin/VE-cadherin. Data are shown as means ± SD of at least three independent experiments and were analyzed by one-way ANOVA followed by a post hoc Dunnett’s test. * *p* ≤ 0.1, ** *p* ≤ 0.01, *** *p* ≤ 0.001, **** *p* ≤ 0.0001 vs. control group.

We first evaluated the effects of murine sAβO42 on endothelial properties in the bEnd.3 monolayer. Exposure of <5 µM murine sAβO42 (12–72 h) had a negligible effect on cell viability in bEnd.3 cells (Figure S1a). At higher concentrations (20, 40 μM), cytotoxicity and reactive oxygen species (ROS) generation were moderately increased, as assessed with MTT and DCFDA assays, respectively (Figure 1B,C). In addition, the effects of sAβO42 on endothelial barrier properties were evaluated. Exposure of sAβO42 did not affect barrier tightness or paracellular diffusion except at a very high concentration (40 µM), as evaluated by transendothelial electrical resistance (TEER) and endothelial permeability to 10 kDa FITC-conjugated dextran at the time points examined (24 h) (Figure 2A,B). We further determined the expression of endothelial junctional proteins in bEnd.3 cells exposed to murine sAβO42. bEnd.3 is proven as a useful tool for studying BBB protein expression under normal and pathological conditions, as it expresses classic BBB proteins (including ZO-1, claudin-5, and P-gp) and responds to inflammatory stimuli [24], although the bEnd.3 monolayer might lack sufficient barrier tightness [24]. Meanwhile, the bEnd.3 cell line is also a common tool for studying the mechanism underlying endothelial dysfunction [25,26]. The expression of ZO-1 and claudin-5 (tight junction (TJ) proteins) in bEnd.3 was significantly reduced by sAβO42 (20 µM for 24 h), as shown by immunofluorescent intensity (IF) (Figure 2C and Figure S1b) and western blot (Figure 3A), in which protein levels of ZO-1 and claudin-5 showed moderate reduction by sAβO42 (10 µM for 24 h). sAβO42 decreased VE-cadherin protein levels significantly (Figure 3A) and increased VE-cadherin-Y731 phosphorylation (Figure 3C), indicating destabilization of monolayer integrity. The protein levels of P-glycoprotein (P-gp or ABCB1) and breast cancer resistance protein 2 (BCRP1), or called ATP binding cassette subfamily G member 2 (ABCG2) (two major BBB transporters) were also significantly reduced by sAβO42 (10–20 µM for 24 h) (Figure 3A). We further demonstrated that sAβO42 reduced constitutive endothelial nitric oxide synthase (eNOS) activity, as indicated by a reduction in the ratio of phosphorylated-eNOS/total-eNOS (Figure 3B). Moreover, sAβO42 (10–40 µM) dose-dependently upregulated matrix metalloproteinase-9 (MMP-9) and decreased basal intercellular adhesion molecular-1 (ICAM-1) protein levels (Figure 3B). ICAM-1 is constitutively expressed on vascular endothelial cells. MMP-9 is known for its ability to induce TJ degradation and proteolytic cleavage of ICAM-1 to form soluble ICAM-1 in the plasma. Taken together, our data indicated that murine sAβO42 might disrupt junctional stability and endothelial homeostasis, possibly via the reduction of eNOS activity and the upregulation of MMP-9 expression.

### 2.3. Resting C8-D1A Releases Soluble Factors That Enhance bEnd.3 Barrier Properties 

**Figure 4:**
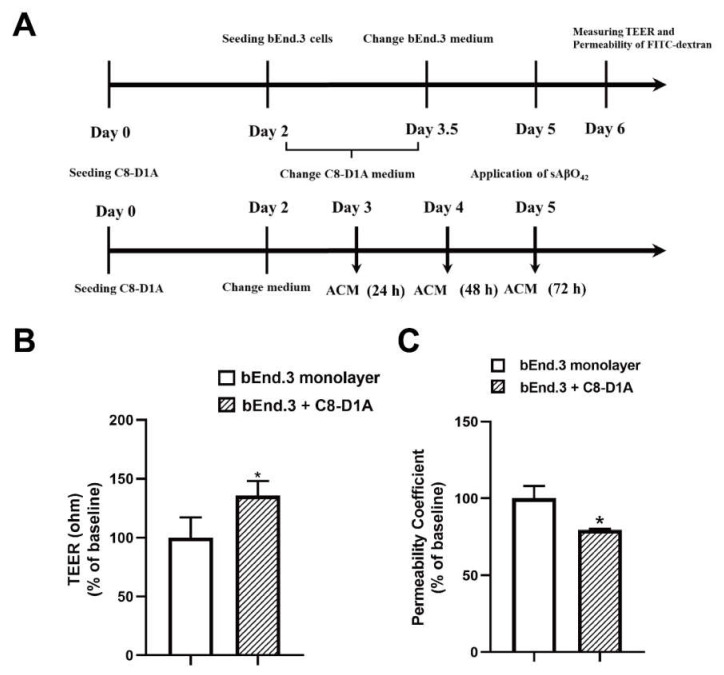
C8-D1A co-culture or astrocyte-conditioned medium (ACM) enhanced bEnd.3 endothelial barrier characteristics. (**A**) Schematic illustration of the preparation of the co-culture and ACM. The day when C8-D1A was defined was day zero (day 0). (**B**) TEER value and (**C**) permeability of FITC-dextran across the bEnd.3 monolayer and bilayer of co-culture system (C8-D1A + bEnd.3). Data are shown as means ± SD of at least three independent experiments and were analyzed by one-way ANOVA followed by a post hoc Dunnett’s test. * *p* ≤ 0.1 vs. control group.

**Figure 5:**
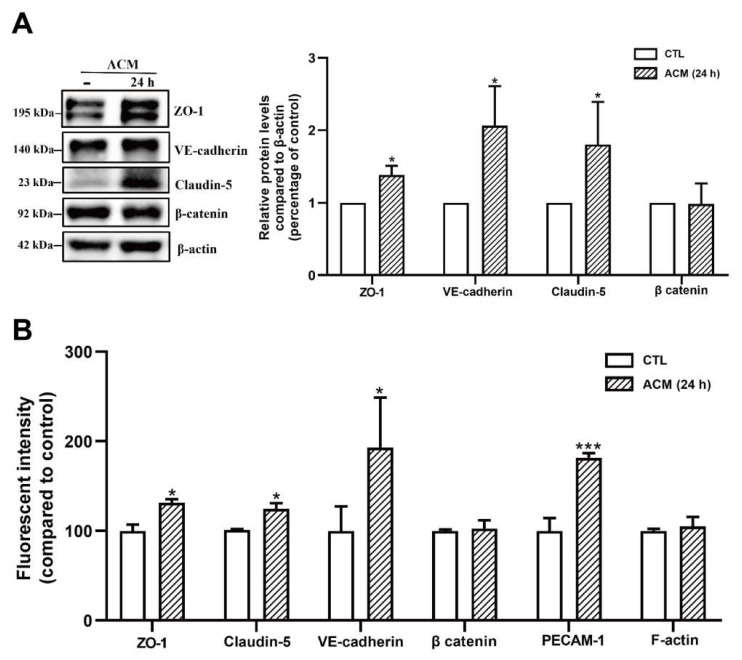
C8-D1A co-culture or astrocyte-conditioned medium (ACM) upregulated the levels of junctional proteins in bEnd.3 cells. (**A**) The protein expression level of junctional proteins ZO-1, VE-cadherin, claudin-5, and β-catenin. (**B**) Fluorescent intensity of junctional proteins ZO-1, claudin-5 and VE cadherin, β catenin, PECAM-1, and cytoskeleton protein F-actin after the treatment of ACM (24 h) for 24 h. Data are shown as means ± SD of at least three independent experiments and were analyzed by one-way ANOVA followed by a post hoc Dunnett’s test. * *p* ≤ 0.1, *** *p* ≤ 0.001 vs. control group.

**Figure 6:**
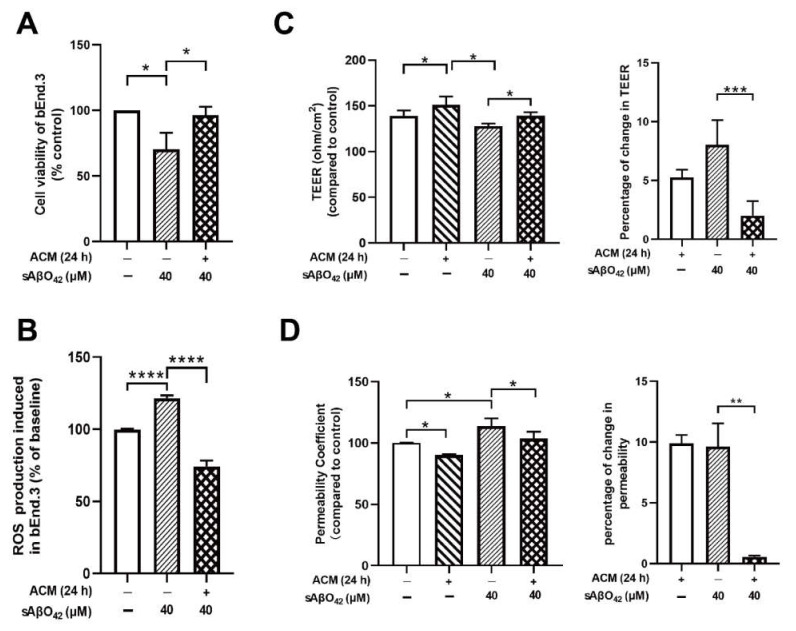
Resting ACM protected cell survival and barrier function of the bEnd.3 monolayer against sAβO42 stimulation. (**A**) Cell viability and (**B**) ROS generation in bEnd.3. (**C**) TEER value and (**D**) permeability of FITC-dextran across the bEnd.3 monolayer. Data are shown as means ± SD of at least three independent experiments and were analyzed by one-way ANOVA followed by a post hoc Dunnett’s test and a post hoc Tukey’s test. * *p* ≤ 0.1, ** *p* ≤ 0.01, *** *p* ≤ 0.001, **** *p* ≤ 0.0001 vs. control group or between two compared groups.

**Figure 7:**
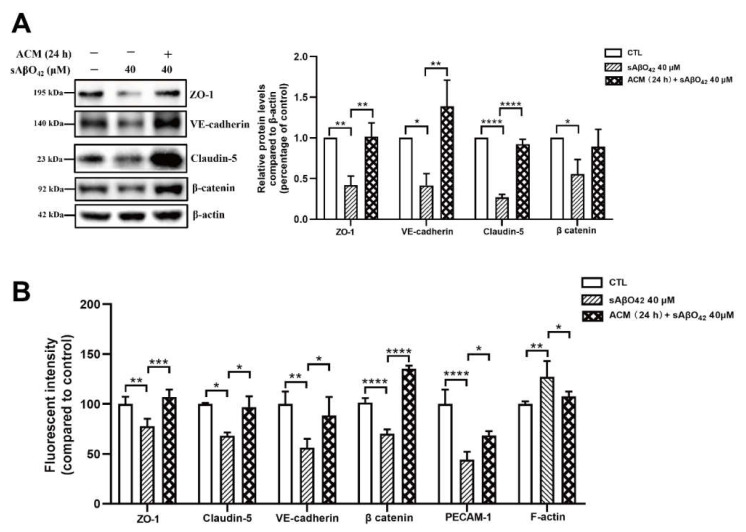
Resting ACM protected normal expression levels of junctional proteins in bEnd.3 cells against sAβO42 stimulation. (**A**) The protein expression level of junctional proteins ZO-1, VE-cadherin, claudin-5, and β-catenin in bEnd.3 cells. (**B**) Fluorescent intensity of junctional proteins ZO-1, claudin-5, VE-cadherin, β-catenin, PECAM-1, and cytoskeleton protein F-actin in bEnd.3 cells. Data are shown as means ± SD of at least three independent experiments and were analyzed by one-way ANOVA followed by a post hoc Dunnett’s test and a post hoc Tukey’s test. * *p* ≤ 0.1, ** *p* ≤ 0.01, *** *p* ≤ 0.001, **** *p* ≤ 0.0001 vs. control group or between two compared groups.

**Figure 8:**
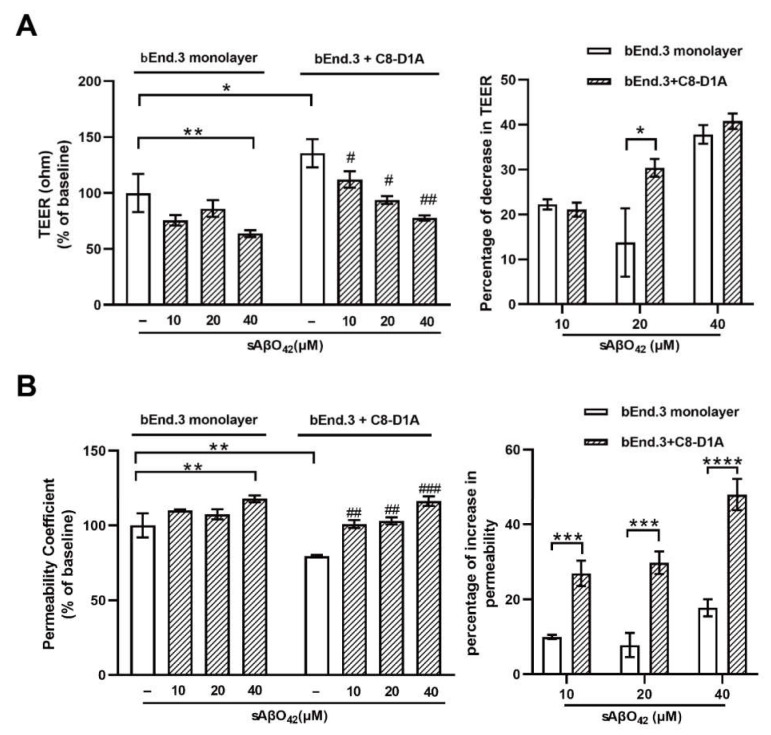
Barrier disruptions induced by murine sAβO42 were enhanced in the bEnd.3 + C8-D1A co culture. (**A**) TEER value and permeability of (**B**) FITC-dextran across the bEnd.3 monolayer and bilayer of the bEnd.3 + C8-D1A co culture system. Data are shown as means ± SD of at least three independent experiments and were analyzed by one-way ANOVA followed by a post hoc Dunnett’s test. * *p* ≤ 0.1, ** *p* ≤ 0.01, *** *p* ≤ 0.001, **** *p* ≤ 0.0001 vs. control group. # *p* ≤ 0.1, ## *p* ≤ 0.01, ### *p* ≤ 0.001 vs. control group in the bEnd.3 + C8-D1A system.

Astrocyte co-culture or astrocyte-conditioned medium (ACM) has been demonstrated to maintain and induce endothelial barrier characteristics, suggesting that astrocyte-derived soluble factor is responsible for endothelial cells developing BBB properties [24]. We further investigated the contribution of astrocytes in our model by using a bEnd.3 + C8-D1A co-culture and ACM derived from C8-D1A. The co-culture was prepared using a non-contact filter insert. Resting ACM was collected from C8-D1A culture at three different time points (24, 48, and 72 h) (Figure 4A). In the absence of sAβO42, the C8-D1A co-culture improved the barrier properties of the bEnd.3 monolayer, as reflected by a higher TEER and lower FITC-dextran permeability (Figure 4B,C). Similarly, resting ACM significantly upregulated the protein expression levels of ZO-1, claudin-5, and VE-cadherin, as shown by western blots (Figure 5A) and immunofluorescence staining (Figure 5B). β-catenin was not affected by resting ACM, as shown by western blotting (Figure 5A) and immunofluorescent staining (Figure 5B). The fluorescent intensity of platelet and endothelial cellular adhesion molecule 1 (PECAM-1) was increased after ACM treatment, which may indicate an increased number of bEnd.3 cells. The expression level of F-actin was not altered significantly, suggesting that the level of cytoskeleton protein was not affected by resting ACM (Figure 5B). We observed that resting ACM collected at 24 h showed the best effect compared to 48 and 72 h (Figure S2a), possibly due to accumulated stress in astrocytes with a longer culturing time. Moreover, resting ACM protected bEnd.3 against sAβO42 challenge, as reflected by reduced cell death and ROS generation (Figure 6A,B), increased TEER, decreased permeability (Figure 6C,D), and reversed junctional protein downregulation, as evaluated by western blots (Figure 7A) and immunofluorescence staining (Figure 7B and Figure S2b). These data show that resting ACM protected bEnd.3 against sAβO42 challenge, as reflected by reduced cytotoxicity and ameliorated TJ and adherens junction (AJ) downregulation. In contrast, endothelial injuries induced by sAβO42 were aggravated in co-culture, as shown in the next section (Figure 8).

### 2.4. Murine sAβO42 Stimulates C8-D1A to Release a Deleterious Factor That Disrupts Endothelial Integrity and Basal eNOS and VEGFR2 Activities in bEnd.3 Cells 

**Figure 9:**
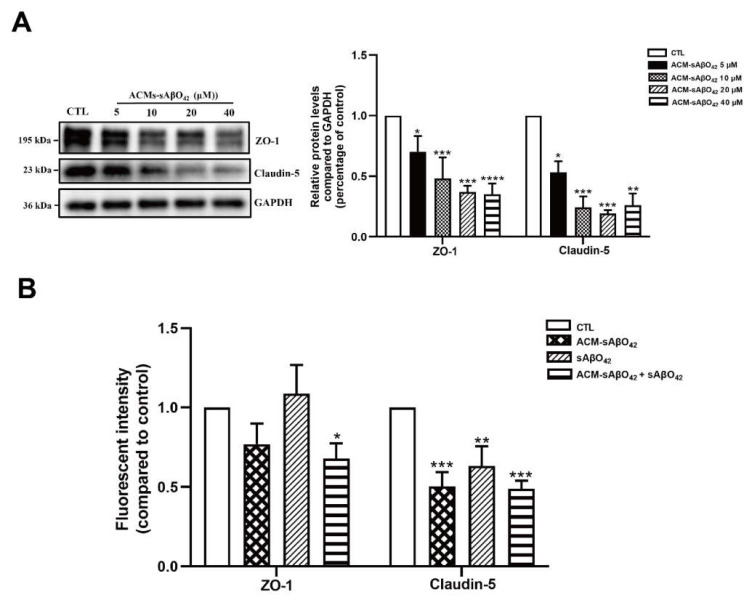
(**A**) Astrocyte secretion stimulated by sAβO42 (ACM-sAβO42) induced significant downregulation of the protein levels of ZO 1 and claudin-5 in a dose-dependent manner. The protein expression level of ZO-1 and claudin-5 upon the exposure of ACM-sAβO42 (5–40 µM for 24 h). (**B**) Fluorescent intensity of junctional proteins ZO-1 and claudin-5 in bEnd.3 cells induced by sAβO42 (10 µM) and ACM-sAβO42 (10 µM). Data are shown as means ± SD of at least three independent experiments and were analyzed by one-way ANOVA followed by a post hoc Dunnett’s test. * *p* ≤ 0.1, ** *p* ≤ 0.01, *** *p* ≤ 0.001, **** *p* ≤ 0.0001 vs. control group.

**Figure 10:**
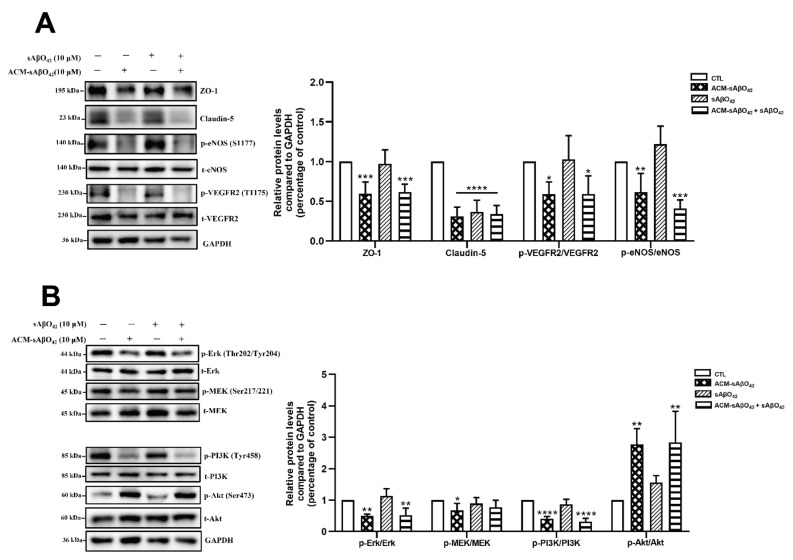
Effects of ACM sAβO42 and exogenous sAβO42 (alone or in combination) on endothelial junctions, eNOS, and vascular endothelial growth factor 2 (VEGFR2) pathways in bEnd.3 cells. (**A**) The protein expression levels of ZO-1, claudin-5, and the ratio of protein expression levels of p-VEGFR2/VEGFR2 and p-eNOS/eNOS in bEnd.3 cells upon the exposure of ACM-sAβO42 (10μM) and sAβO42 (10 μM) for 24 h. (**B**) The ratio of protein expression levels of p-Ras/Raf/Mitogen-activated protein kinase/ERK kinase (MEK)/t-MEK, p-extracellular-signal-regulated kinase (Erk)/t-Erk, p-phosphoinositide 3-kinase (PI3K)/t-PI3K, and p-serine/threonine kinase Akt (Akt)/t-Akt. Data are shown as means ± SD of at least three independent experiments and were analyzed by one-way ANOVA followed by a post hoc Dunnett’s test. * *p* ≤ 0.1, ** *p* ≤ 0.01, *** *p* ≤ 0.001, **** *p* ≤ 0.0001 vs. control group.

Figure 8 showed that murine sAβO42 (10–40 µM) induced more pronounced injuries in the co-culture system (bEnd.3 + C8-D1A) compared to the endothelial monolayer. The barrier integrity of the co-culture was disrupted by sAβO42 in a dose-dependent manner, as shown by a significant decrease in TEER value and an increase in the permeability coefficient (Figure 8A,B). A previous study reported that Aβ-treated astrocytes released soluble factors that contributed to Aβ-induced BBB damage in a co-culture model of human brain endothelial and astrocyte cell lines [13]. Therefore, we further prepared ACM-sAβO42 by exposing C8-D1A to murine sAβO42 (5–40 µM for 24 h). In line with this, we showed that ACM-sAβO42 induced pronounced disruption in TJ protein levels in bEnd.3 and significantly downregulated ZO-1 and claudin-5 in an Aβ-dose-dependent manner, as evaluated by western blot (Figure 9A). However, exogenous sAβO42 (10µM) produced fewer injuries, as indicated by immunofluorescent staining and western blot results for ZO-1 and claudin-5 (Figure 9B, Figure 10A and Figure S2c). Furthermore, ACM-sAβO42 significantly reduced the levels of p-eNOS/eNOS and p-vascular endothelial growth factor receptor 2 (VEGFR2)/VEGFR2 without a reduction in total protein levels (Figure 10A), indicating a reduction in basal eNOS and VEGFR2 activities. Co-treatment of ACM-sAβO42 and exogenous sAβO42 did not produce additive effects on TJ protein downregulation (Figure 9B and Figure 10A), as well as no significant further reduction in p-eNOS/eNOS and p-VEGFR2/VEGFR2 (Figure 10A), suggesting that these endothelial activities were highly prone to perturbation induced by ACM-sAβO42. These data show that, in contrast to resting conditions, astrocytes stimulated by sAβO42 induced pronounced injuries to the endothelium. It is reported that Aβ-treated astrocytes released soluble factors, such as proinflammatory cytokines (e.g., tumor necrosis factor alpha (TNF-α) and interleukin 6 (IL-6) [27], interleukin 1β (IL-1β) [28], monocyte chemoattractant protein-1 (MCP-1) [29], and interferon-inducible protein-10 (IP-10) [30]) and vasoactive agents (e.g., vascular endothelial growth factor (VEGF) [13], matrix metalloproteinases (MMPs) [31], nitrox oxide (NO), and ROS [32]), therefore it is likely that ACM-sAβO42 would contain a mixture of inflammatory cytokines, chemokines, and vasoactive agents, which contributed to Aβ-induced BBB damage.

Constitutive and continuous expressions of vascular endothelial growth factor (VEGF) and its receptor vascular endothelial growth factor receptor 2 (VEGFR2) by endothelial cells are needed to maintain vascular integrity and cellular viability, and their reduction or inactivation results in endothelial cell demise [33]. Therefore, we further evaluated the effects of ACM-sAβO42 on the downstream signaling of the VEGFR2 pathway. Our data showed that p-Ras/Raf/Mitogen-activated protein kinase/ERK kinase (MEK)/MEK and p- extracellular-signal-regulated kinase (Erk)/Erk were significantly reduced by ACM-sAβO42 (Figure 10B), indicating suppression of the pro-survival MEK/ERK pathway [34]. ACM-sAβO42 also decreased p-phosphoinositide 3-kinase (PI3K)/PI3K but increased p-serine/threonine kinase Akt (Akt)/Akt (Figure 10B). Although PI3K activity is the major mode of Akt activation, other kinases have been shown to activate Akt directly in response to inflammation or DNA damage even if PI3K activity is inhibited [35]. The Akt pathway has a critical role in endothelial maintenance, and it is frequently dysregulated in vascular pathologies.

**Figure 11:**
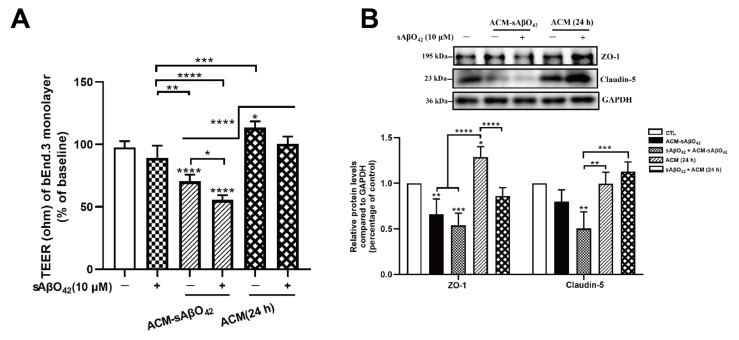
Resting ACM and ACM-sAβO42 had opposite effects on endothelial properties and protein expression of junctional proteins. (**A**) TEER value of the bEnd.3 monolayer after the treatment of ACM sAβO42 (10 µM for 24 h), resting ACM (24 h), or exogenous sAβO42 alone or in combination. (**B**) The protein expression levels of tight junction proteins ZO-1 and claudin-5. Data are shown as means ± SD of at least three independent experiments and were analyzed by one-way ANOVA followed by a post hoc Dunnett’s test and a post hoc Tukey’s test. * *p* ≤ 0.1, ** *p* ≤ 0.01, *** *p* ≤ 0.001, **** *p* ≤ 0.0001 vs. control group or between two compared groups.

**Figure 12:**
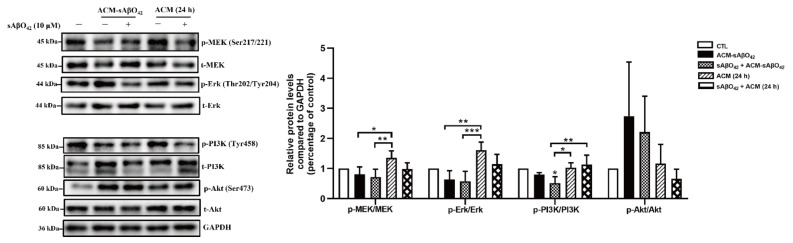
Resting ACM and ACM-sAβO42 had opposite effects on the VEGFR2 downstream pathways. The expression levels of proteins (pMEK/MEK, pErk/Erk, pPI3K/PI3K, and pAkt/Akt) in VEGFR2 downstream pathways in bEnd.3 cells. Data are shown as means ± SD of at least three independent experiments and were analyzed by one-way ANOVA followed by a post hoc Dunnett’s test and a post hoc Tukey’s test. * *p* ≤ 0.1, ** *p* ≤ 0.01, *** *p* ≤ 0.001 vs. control group or between two compared groups.

We further compared the endothelial alterations in bEnd.3 cells treated with ACM-sAβO42 and resting ACM (Figure 11 and Figure 12). As shown in Figure 11A, ACM-sAβO42 (10 µM for 24 h) reduced TEER by 30% compared to resting ACM, and the addition of exogenous sAβO42 (10 µM) further enhanced the TEER reduction to 50%. Figure 11B and Figure 12A show the western blots of proteins. It was observed that resting ACM increased the levels of ZO-1, claudin-5, p-MEK/MEK, and p-ERK/ERK in bEnd.3 cells compared to the control, indicating that soluble factors derived from astrocytes promoted TJ protein expression and pro-survival MEK/ERK signaling. ACM-sAβO42 significantly reduced the protein levels of ZO-1, claudin-5, p MEK/MEK, p ERK/ERK, and p-PI3K/PI3K approximately by 50%, while p-Akt/Akt was increased compared to resting ACM (Figure 11B and Figure 12A). Exogenous sAβO42 alone produced fewer toxic effects, and the combination of ACM-sAβO42 and exogenous sAβO42 produced the most disruptions. Taken together, our data showed that murine sAβO42 produced its toxic effects mainly through stimulating astrocytes to release deleterious soluble factors that caused significant dysfunction of the VEGFR2 downstream signaling pathway, including decreased activity of eNOS and VEGFR2, reduction in the pro-survival MEK/ERK pathway and dysregulation of PI3K/Akt signaling in endothelial cells. 

## 3. Discussion

In the present study, we aimed to evaluate the direct effects of murine Aβ on the BBB cellular components and their interactions using murine endothelial and astrocyte cell lines (bEnd.3 and C8-D1A) *in vitro*. The vascular effects of Aβ are dependent on the type of peptide (e.g., Aβ1–40 or Aβ1–42), aggregation status (e.g., soluble monomer, dimer, oligomer, and insoluble protofibril and fibrillary aggregates) and concentration [9]. High-molecular weight (HMW) Aβ42 oligomers (>70 kDa) were identified as the major soluble species in AD patients [36]. Our preparation of murine Aβ42 oligomers (sAβO42) consisted predominantly of HMW oligomers (>70 kDa) (Figure 1). Mouse Aβ has been reported to be less aggregable and less toxic than human Aβ due to amino acid substitutions at positions 5, 10, and 13 [37]. In line with this, our data showed that exogenous administration of murine sAβO42 (10–40 µM) caused modest endothelial cell death and intracellular oxidative stress (Figure 1). More interestingly, sAβO42 induced more pronounced injuries on endothelial barrier properties with reduced protein levels of endothelial TJ and AJ (ZO-1, claudin-5, and VE-cadherin) and BBB transporters (P-gp and BCRP), with a concomitant decrease in TEER and increased permeability. There was also an increase in VE-cadherin-Y731 phosphorylation (Figure 3C), indicating AJ dissociation and barrier destabilization. We observed that several constitutively expressed proteins that are essential for endothelial maintenance, such as p-eNOS (Figure 3B) and ICAM-1 (Figure 3B), had reduced activities after sAβO42 exposure, indicating the aberration of endothelial functions. In addition, there was an upregulation of MMP-9, which is known for its ability to induce TJ degradation. It is reported that increased levels of MMP-2, MMP-3, and especially MMP-9 in endothelial cells [38] and MMP secretion by astrocytes [39] and pericytes [40] are widely observed in BBB disruption. Knocking out the *MMP-9* gene in an ischemic mouse model showed significant BBB protection and neuroprotection [41,42]. Taken together, murine sAβO42 (10–40 µM) directly induced barrier injuries and disturbed endothelial functions but was less toxic than human Aβ42 (the toxic concentration of human Aβ42 in human or rat cell lines is reported to be around 1–20 µM, as reviewed in [9]).

We further investigated the contribution of astrocytes in our model with a co-culture (bEnd.3 + C8 D1A) and ACM derived from C8-D1A. We observed that in the absence of sAβO42, the co-culture and ACM improved the barrier properties of the bEnd.3 monolayer, as reflected by a higher TEER value, lower permeability, and upregulated expression of TJ and AJ proteins (Figure 4 and Figure 5). In contrast, endothelial injury induced by sAβO42 was aggravated in the co-culture system when compared to that in the endothelial monolayer (Figure 8). 

Astrocytes are known for their highly secretory nature and they release either endothelial-promoting or endothelial-disrupting factors depending on the surrounding stimuli [43,44]. Astrocyte activation is observed in various transgenic AD mouse models, including *PS1V97L* [45], Tg2576 [46], 5 × FAD [47], 3 × Tg-AD mice [48], as well as AD models established by intracerebroventricular or hippocampal injection of Aβ species (Aβ42 oligomers [27,49], Aβ25–35 [50]). Concomitant activations of nuclear factor-κB (NF-κB) [49], (NLR family Pyrin Domain Containing 3) NLRP3/caspase-1 [51], and calcineurin (CN)/(nuclear factor of activated T cells) NFAT pathways [47] are observed to accompany astrocyte activation, which induces the production of secretions from astrocytes, causing neuroinflammation. In addition, increased release of proinflammatory cytokines (TNF, IL-6 [27], IL-1β [28], MCP-1 [29], IP-10 [30]), VEGF [13], MMPs [31], NO, and ROS [32] were observed in astrocytes that were stimulated by Aβ42 oligomers *in vitro*. Therefore, in our present study, it is likely that ACM-sAβO42 would contain a mixture of inflammatory cytokines and chemokines and vasoactive agents, such as VEGF, MMPs, NO, and ROS. Furthermore, vicious secretions from astrocytes have been shown to induce BBB disruption *in vivo* (reviewed in [44]). In *in vitro* experiments, two recent studies using human cell lines showed that astrocytes stimulated by Aβ42 secreted VEGF and induced BBB damage via upregulation of endothelial MMP-9 [13,39]. In line with this, we demonstrated that ACM-sAβO42 (for 24 h) (prepared by exposing C8-D1A to murine sAβO42) induced pronounced reductions in ZO-1 and claudin-5 (Figure 9A) in a dose-dependent manner. Moreover, ACM-sAβO42 exposure caused significant reductions in p-eNOS/eNOS and p-VEGFR2/VEGFR2 (Figure 10). The activities of eNOS and VEGFR2 are essential for endothelial maintenance [52]. Our results suggested that these endothelial homeostatic pathways might be highly sensitive and prone to perturbation under the environment of Aβ pathology. The addition of exogenous sAβO42 in combination with ACM-sAβO42 did not further reduce p-eNOS/eNOS or p-VEGFR2/VEGFR2 (Figure 10A), suggesting that astrocyte-secreted soluble factors were the main culprit of endothelial perturbation. In agreement, the phosphorylation of VEGFR2 downstream signal proteins, including p-MEK, p-ERK, and p-PI3K, was significantly reduced by ACM-sAβO42 exposure (Figure 10B). Since MEK/ERK and PI3K/Akt are essential signaling pathways for endothelial survival, proliferation, migration, and permeability [52,53,54], disturbances in these pathways might lead to endothelial dysregulation. 

It is interesting to note that, under normal circumstances, the VEGF/VEGFR2/MEK/ERK pathway is also responsible for downstream eNOS phosphorylation, which could lead to hyperpermeability in response to inflammation [34,55]. However, in the current study, it is likely that VEGFR2 signaling and downstream pathways are dysregulated by endothelial-damaging factors (such as inflammatory cytokines and MMPs) secreted from stimulated astrocytes. Indeed, MMPs have been reported to mediate VEGFR2 degradation and lead to cerebral vascular dysfunction and neuroinflammation [56]. Inflammatory cytokines could impose a wide variety of effects on VEGFR2 signaling, such as the transcription of endothelial cytokine receptors [57], activation of a vascular proinflammatory response [58], and cytokine-induced downregulation of eNOS expression and activity [59]. Furthermore, we observed that sAβO42 alone, or exogenous sAβO42 added to ACM-sAβO42, did not alter endothelial VEGFR2 signaling. 

Taken together, our results suggested that sAβO42 stimulated astrocytes (possibly via the receptor for advanced glycation end products (RAGE) on astrocytes [60]) to produce deleterious factors, which likely included inflammatory cytokines, VEGF, and MMPs. These deleterious factors possibly disturbed endothelial homeostasis via dysregulating endothelial VEGFR2 signaling and related downstream pathways, including MEK/ERK and PI3K/Akt, and thus disrupted endothelial survival and BBB maintenance.

## 4. Materials and Methods

### 4.1. Materials and Reagents

Dulbecco’s Modified Eagle Medium (DMEM), fetal bovine serum (FBS), penicillin–streptomycin (P/S), phosphate-buffered saline (PBS), 0.25% (*w*/*v*) trypsin/1mM EDTA were purchased from (Gibco, Invitrogen, Grand Island, NY, USA). Dimethyl sulfoxide (DMSO), MTT [3-(4,5-dimethylthiazol-2-yl)-2,5-diphenyltetrazoliumbromide], FITC-conjugated dextran (1 mg/mL of fluorescein isothiocyanate-dextran, molecular: 40 kDa) (Sigma-Aldrich, St Louis, MO, USA) and amyloid beta 42 were acquired from ChinaPeptides Co., Ltd. Purified water was prepared with a Milli-Q purification system from Millipore (St Louis, MO, USA). VE-Cadherin antibody (Cat. No: 2500S), β-actin antibody (Cat. No: 4970S), VEGFR2 antibody (Cat. No:2472S), Phospho-VEGFR2 antibody (Cat. No:2478S), total eNOS antibody (D9A5L) (Cat. No: 32027S), PI3K (Cat. No: 4257), Phospho-PI3K antibody (Cat. No:4228S), Akt antibody (Cat. No:4691S), Phospho-Akt antibody (Cat. No:4060S), MEK antibody (Cat. No:9122L), Phospho-MEK antibody (Cat. No:9121S), ERK antibody (Cat. No: 9102S), Phospho-ERK antibody (Cat. No: 9101S), Anti-rabbit IgG, HRP-linked antibody (Cat. No: 7074S), Anti-rabbit IgG (H+L) (Alexa Fluor^®^ 488 Conjugate) (Cat. No: 4412S), Anti-rabbit IgG (H+L) (Alexa Fluor^®^ 594 Conjugate) (Cat. No: 8889S), and DY-554 Phalloidin (Cat. No: 13054S) were all purchased from Cell Signaling Technology (Beverly, MA, USA). Claudin-5 antibody (Cat. No:34–1600) and ZO-1/TJP1 Antibody (Cat. No:40-2200), Phospho-eNOS (Ser1177) polyclonal antibody (Cat. No: 9571S), ICAM-1(Cat. No: MA5407), and RAT anti-MOUSE CD31/PECAM-1 (L11344) antibody were purchased from Thermo Fisher Scientific (Waltham, MA, USA). P-glycoprotein antibody (Cat. No: 22336-1-AP) and MMP-9 (Cat. No: 10375-2-AP) were purchased from Proteintech Group, Inc (Rosemont, PA, USA). BCRP antibody (Cat. No: AP50949) was purchased from Abcepta (China). Immortalized mouse brain endothelial cells (bEnd.3) and mouse astrocyte cells (C8-D1A) were obtained from American Type Culture Collection (ATCC, Manassas, VA, USA).

### 4.2. Cell Culture

bEnd.3 cells (CRL-2299) and C8-D1A (CRL-2541) were incubated in Dulbecco’s Modified Eagle’s Medium (DMEM) supplemented with 10% FBS and 1% penicillin–streptomycin (P/S). bEnd.3 cells and C8-D1A cells were authenticated by evaluating the expressions of specific biomarkers (CD31 for bEnd.3 and GFAP for C8-D1A). Both cells showed typical morphologies under the microscope. Cells were incubated at 37 °C in a humidified atmosphere with 5% CO2. Confluent cells were passaged by the trypsin (0.25%)–EDTA (0.02%) solution at a split ratio of 1:4–1:5. We generated the monolayer model and co-culture model referred to in the protocols suggested by Hawkins et al., and Gaillard et al. [36,61]. bEnd.3 cells were seeded on the surface of the membrane in Polyester Transwell inserts (0.4 μM pore size, 6.5 mm diameter, 24 well; Corning Costar, Kennebunk, ME, USA) at a density of 4 × 104/cm^2^ to generate the monolayer model. For the generation of the co-culture model, C8-D1A (5 × 103/cm^2^) was seeded on the outer side of the insert pre-coated with 100 μL poly-L-Lysine (10.0 mg/mL). bEnd.3 cells (4 × 104/cm^2^) were seeded in the inner side of the insert membrane after the 48 h experiment was performed after the cells came to confluence for about 3 days for the monolayer and 5 days for the co-culture model [53]. As controls, bEnd.3 and C8-D1A were cultured alone in different wells. All cells were cultured with a daily change of medium before analysis.

### 4.3. Preparation of Murine Amyloid Beta (42) (Aβ42) Oligomers

Soluble oligomeric Aβ42 was prepared as described in Stine et al., paper [23]. Briefly, lyophilized synthetic murine Aβ42 was dissolved in 100% HFIP (hexafluoroisopropanol) (Sigma-Aldrich, St Louis, MO, USA), then HFIP was evaporated and the dry peptide was stored at −20°C. For oligomeric conditions, the peptide was first resuspended in DMSO to 10 mM and diluted in cell culture medium to bring the peptide to the required concentration of 100 μM and incubated at 4 °C for 24 h before use, and then the solution would be diluted into required concentrations (5–40 μM).

### 4.4. Preparation of Astrocyte-Conditioned Medium (ACM)

We prepared an astrocyte-conditioned medium (ACM) based on the protocols of several papers [62,63,64]. C8-D1A cells were cultured in DMEM at 37 °C in a humidified incubator containing 5% CO_2_. The collection timepoint of ACM is parallel with the key timeline of the co-culture system establishment. C8-D1A cells was seeded at density of 5 × 10^3^/cm^2^. ACM was collected on the third day after seeding. TEER and permeability were measured three days after bEnd.3 cell seeding. Therefore, ACM was collected at 24, 48, and 72 h. The collected ACM was then centrifuged at 1000*g* for 10 min. Supernatants were collected and stored at –20 °C. ACM-Aβ was collected after 24 h treatment with a certain concentration of sAβO42.

### 4.5. Cell Viability Assay

The cell viability was measured with an MTT assay, as described elsewhere. bEnd.3and C8-D1A cells were trypsinized and respectively seeded at the density of 3 × 104/cm^2^ and 1.5 × 104/cm^2^ in 96-well plates. After 24 h incubation, cells were treated with sAβO42. Then, the medium was discarded and cells were incubated for 4 h at 37 °C in 0.5 mg/mL MTT solution. The solution was then replaced by 100 μL DMSO to dissolve the violet formazan crystals in intact cells. The absorbance was measured at a wavelength of 570 nm by a SpectraMaxR M5 Multi-Mode Microscope Reader. (San Jose, CA, USA) Cell viability was shown as a percentage of the vehicle control.

### 4.6. ROS Assay

The level of intracellular reactive oxygen species (ROS) was quantified using the Reactive Species Assay Kit (Beyotime, China) and the experiment was performed according to the manufacturer’s instructions. DCFDA was oxidized by reactive oxygen species in viable cells to 2′,7′-dichlorofluorescein (DCF), which is highly fluorescent at 530 nm. Cells were washed three times with PBS and then incubated with DCFDA (diluted to a final concentration of 10 μM) for 30 min at 37 °C in the dark. After washing three times with PBS, cells were recovered with 10% (*v*/*v*) FBS medium and incubated at 37 °C for 45 min. The fluorescent intensity of cells in the 6-well were detected with a SpectraMaxR M5 Multi-Mode Microscope Reader at an excitation length of 492 nm and emission at 517 nm. The level of intracellular ROS was shown as the percentage of the control cells.

### 4.7. TEER Measurement

Trans-endothelial electrical resistance (TEER) was measured using an Epithelial Volt/Ohm Meter (EVOM) with TEER STX 100 electrodes (World Precision Instrument, Sarasota, FL, USA). The TEER value of the blank insert was subtracted from the measured TEER value of the model layer (monolayer or co-culture). Treatments were performed when TEER values were stable. TEER values measured in the bEnd.3 monolayer and bEnd.3 + C8-D1A bilayer were 128 ± 0.5 Ω·cm^2^ and 166 ± 0.5 Ω·cm^2^, respectively, comparable to previously published TEER values measured in *in vitro* mouse endothelial cell models (around 50–300 Ω·cm^2^) [24]. The measurement was performed according to the manufacturer’s instructions.

### 4.8. Permeability of FITC-Conjugated Dextran

Trans-endothelial permeability was measured using FITC-dextran (1 mg/mL of fluorescein isothiocyanate-dextran, molecular mass: 40 kDa) (Sigma Aldrich, St Louis, MO, USA). The method was based on the protocol suggested in Natarajan et al [65]. After maturation of junctions, insert and well were washed with blank medium to remove the color of the medium. An FITC–dextran solution in blank medium was added into the insert and the same column of blank medium in the well. Then, 50 μL of fluorescent solution was collected respectively from the insert and the lower compartment after 6 h treatment. Fluorescence (excitation length: 494 nm, emission length: 520 nm) was evaluated with SpectraMaxR M5 Multi-Mode Microscope Readers. The permeability coefficient was determined using the following formula: P-dextran = (RFUlower/RFUupper) (V) (1/t) (1/A), where RFU is the relative fluorescent units in the upper and lower wells, V is the volume of the bottom well, t is the time that the FITC–dextran was allowed to diffuse, and A is the total surface area of the monolayer (cm^2^). Permeability coefficients were normalized by setting basal cell monolayers to a value of 100%. Permeability coefficients measured in the bEnd.3 monolayer, the C8-D1A monolayer and the bEnd.3 + C8D1A co-culture were 9.446 ± 1.494 μL/h·cm^2^, 11.72 ± 1.434 μL/h·cm^2^, and 6.438 ± 0.3236 μL/h·cm^2^, respectively. (The permeability coefficient for the blank insert was 17.10 ± 0.2273 μL/h·cm^2^)

### 4.9. Immunofluorescence

Immunofluorescent staining was performed according to the standard procedures. bEnd.3 cells were seeded in a 96-well plate. Cell–cell junctions were allowed to establish over 2 days. After treatment of sAβO42 or ACMs, cells were washed with PBS and fixed in 4% PFA for 20 min. Then, cells were permeabilized with PBS containing 0.3% Triton X-100 for 20 min on ice after being washed 3 times with PBS for 5 min. Cells were blocked with 2% BSA in PBS for 1 h at room temperature. Primary antibodies (1:500) were applied in 2% BSA in PBS and incubated overnight at 4 °C. Cells were then washed 3 times with PBS for 5 min. Secondary antibodies (1:1000) conjugated to fluorescent probes (Alexa Fluor 488 Rabbit Anti-Goat IgG (H+L) and Alexa Fluor 594 Rabbit Anti-Goat IgG (H+L)) were applied for 1 h at room temperature. Cells were washed 4 times with PBS for 5 min per wash and images were taken with a IN Cell Analyzer 2000 system (General Electric, Bradenton, FL, USA).

### 4.10. Western Blotting

Western blotting was carried out in accordance with the standard procedures. Cells that were used for western blotting analysis were treated in a 6-well culture plate (Cat No. 30006, SPL Life Sciecnce, Korea) or tissue culture dish (60 mm, Cat No. 0030701100, Eppendorf, Framingham, MA, USA). Cells were then washed with ice cool PBS and lysed for 30 min on ice with RIPA lysis buffer. Cell lysates were centrifuged at 12,500*g* for 20 min at 4 °C. Protein concentrations in the supernatants were measured using the bicinchoninic acid (BCA) assay (Pierce, Rockford, IL, USA)). Equal amounts of protein (30 μg) from different cell groups were electrophoresed on 10% SDS-PAGE and transferred to a polyvinylidene fluoride (PVDF) membranes and were then blocked with 5% non-fat milk. Immunoblot analysis was performed by incubation with primary antibodies (1:1000) at 4 °C overnight. After washing, membranes were incubated for 1h at room temperature with horseradish peroxidase-conjugated goat anti-rabbit IgG (1:2000) (Cat No. A0208, Beyotime, China). Proteins were visualized using an advanced enhanced ECL system (GE Healthcare, Malborough, MA, USA). SDi-quantifications were performed with densitometry analysis using Bio-Rad Image 5.1 Software (Bio-Rad Laboratories, Inc, Irvine, CA, USA).

### 4.11. Statistical Analysis

Each experiment was repeated at least three times independently (*n* ≥ 3). The experimenter in this study was not blind to the study design of the *in vitro* experiments. Statistical analysis was performed using the software GraphPad Prism (version 8.0.2 for Windows, GraphPad Software, San Diego, California, USA). The mean and standard error of the mean (SEM) were compared using one-way ANOVA analysis followed by a post hoc Dunnett’s multiple comparisons test. Statistical significance was defined as: *p < 0.05, significant; **p < 0.01, highly significant; ***p < 0.001 extremely significant (vs. CTL). All experiments were conducted under the standard experimental protocols of the University of Macau (Ethical guidelines of the ICMS, University of Macau).

## 5. Conclusions

In conclusion, we evaluated the vasculotoxicity of murine sAβO42 in monolayer and co-culture models of BBB using mouse brain endothelial cells (bEnd.3) and mouse astrocytes (C8-D1A). Our results showed that murine sAβO42 disrupted endothelial integrity and barrier functions by stimulating astrocytes to release deleterious soluble factors that caused endothelial disruption and dysfunction possibly related to the reduction in eNOS activity and MEK/ERK signaling, both of which have critical roles in endothelial maintenance. Our data further provided important evidence for using aging-dependent mouse models for the study of Aβ pathogenesis in AD.

## Data Availability

The data presented in this study are available in this article and Supplementary Materials. The datasets used and/or analyzed during the current study are available from the corresponding author on reasonable request.

## Supplementary Materials

The following are available online at https://www.mdpi.com/article/10.3390/ijms23031878/s1.
