# Supplementary material for: Murine Beta-Amyloid (1–42) Oligomers Disrupt Endothelial Barrier Integrity and VEGFR Signaling via Activating Astrocytes to Release Deleterious Soluble Factors
Source: Int J Mol Sci. 2022 Feb 7;23(3):1878. doi: 10.3390/ijms23031878 (PMC8836933; doi:10.3390/ijms23031878)
Supplement: Supplementary file 1 [file ijms-23-01878-s001.zip › supplementary files/supplementary figures.pdf]

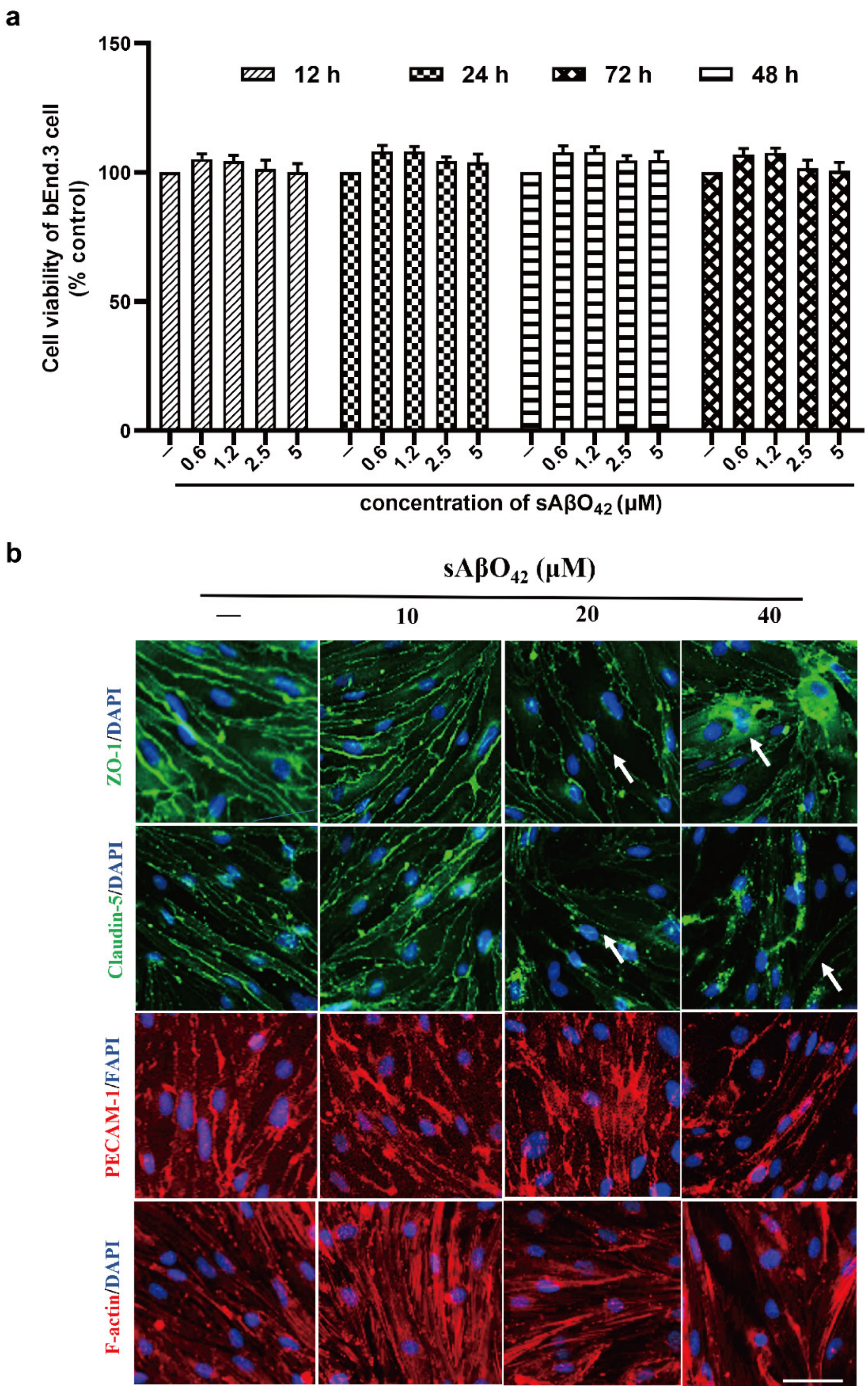

Figure S1. (**a**) sAβO42 (<5μM) showed no toxicity on cell viability of bEnd.3 cells. (**b**) sAβO42 decreased the expressions of junctional proteins in bEnd.3 in a dose-dependent manner. Immunofluorescent staining of tight junction proteins (ZO-1, Claudin-5), adherens junction proteins (VE-cadherin, PECAM-1, β-catenin) and cytoskeleton (F-actin). Scale bar, 3μm. Detected by IN Cell Analyzer 2000 (40×, 21℃) and analyzed by Image J software. Data are shown as mean±SD of three independent experiments and analyzed by one-way ANOVA followed by Tukey's test. *P≤0.1, **P≤0.01, ***P≤0.001 vs. control group.

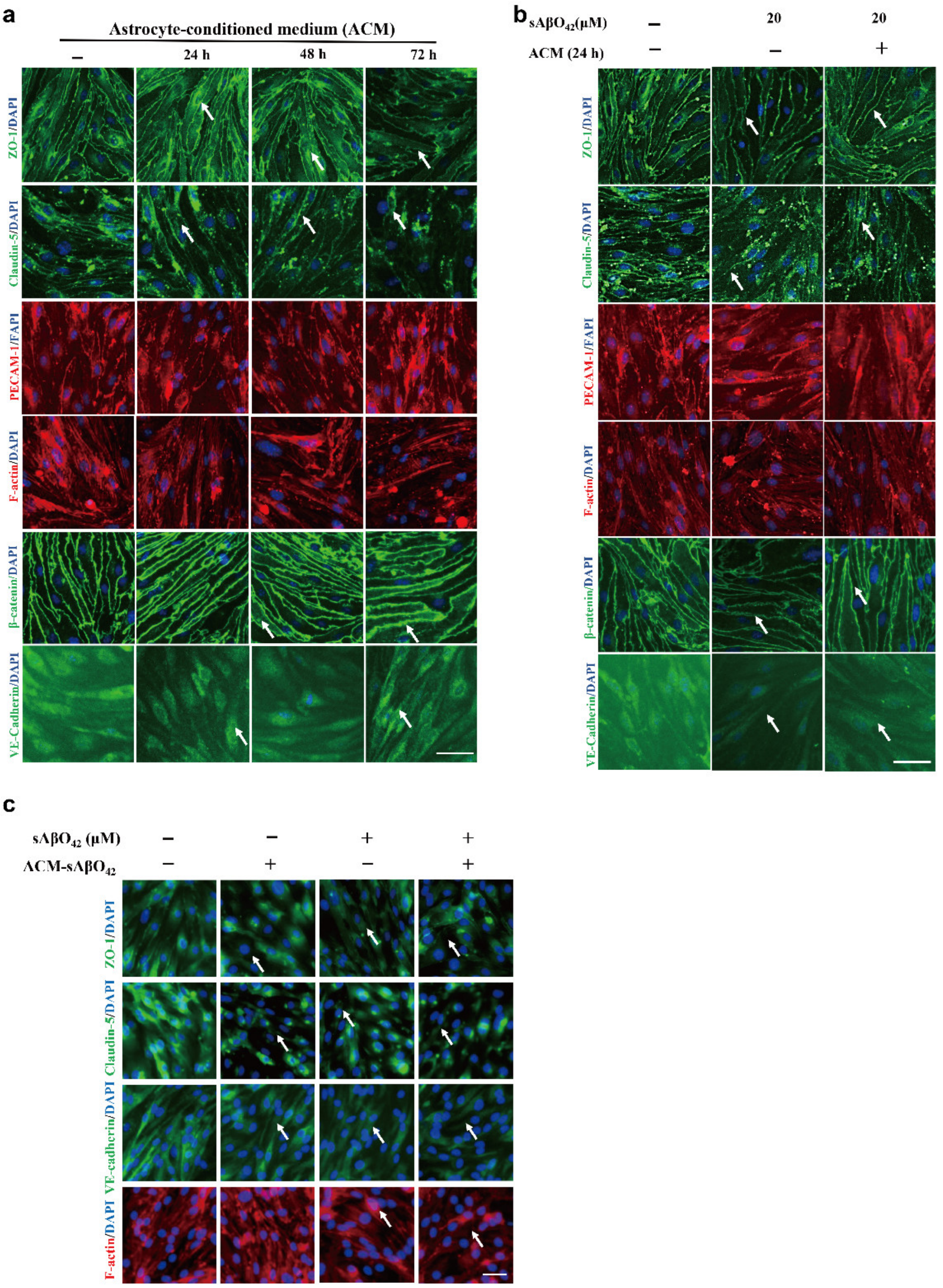

Figure S2. (**a**) Effects of ACM collected at different time points on the expression level and localization of tight junction proteins (ZO-1, Claudin-5), adherens junction proteins (VE-Cadherin, PECAM-1, β-catenin) and cytoskeleton (F-actin). (**b**) Protective effects of ACM (24h) on sAβO42-induced downregulation of junctional proteins in bEnd.3 cells. Immunofluorescent staining of tight junction proteins (ZO-1, Claudin-5), adherens junction proteins (VE-cadherin, PECAM-1, β-catenin) and cytoskeleton (F-actin). **(c)** ACM-Aβ(10μM) resulted in downregulation of ZO-1, Claudin-5, VE-cadherin and F-actin with or without sAβO42(10μM) cotreatment. sAβO42(10μM) induced no loss of ZO-1 or less loss of Claudin-5. Immunofluorescent staining of tight junction proteins (ZO-1, Claudin-5), adherens junction proteins (VE-cadherin) and cytoskeleton (F-actin). Scale bar, 3μm. Detected by IN Cell Analyzer 2000 (40×, 21°C) and Analyzed by Image J software.
